# Effect of Ultra-High-Molecular-Weight Molecular Chains on the Morphology, Crystallization, and Mechanical Properties of Polypropylene

**DOI:** 10.3390/polym13234222

**Published:** 2021-12-01

**Authors:** Takumitsu Kida, Takeyoshi Kimura, Ayaka Eno, Khunanya Janchai, Masayuki Yamaguchi, Yasuhiko Otsuki, Tokutaro Kimura, Tomoaki Mizukawa, Tomoya Murakami, Kazuki Hato, Tomoya Okawa

**Affiliations:** 1 Japan Advanced Institute of Science and Technology, School of Materials Science, 1-1 Asahidai, Nomi 9231292, Japan; s2120012@jaist.ac.jp (T.K.); s2010016@jaist.ac.jp (A.E.); s1910444@jaist.ac.jp (K.J.); m_yama@jaist.ac.jp (M.Y.); 2 Petrochemical and Polymer Science, Faculty of Science, Chulalongkorn University, Pathumwan, Bangkok 10330, Thailand; 3 Packaging and Industrial Materials Laboratory, Prime Polymer Co., Ltd., 3 Chigusa-Kaigan, Ichihara 2990108, Japan; yasuhiko.otsuki@primepolymer.co.jp (Y.O.); tokutaro.kimura@primepolymer.co.jp (T.K.); tomoaki.mizukawa@primepolymer.co.jp (T.M.); tomoya.murakami@primepolymer.co.jp (T.M.); kazuki.hato@primepolymer.co.jp (K.H.); tomoya.okawa@primepolymer.co.jp (T.O.)

**Keywords:** ultra-high molecular weight component, rheological property, crystallization behavior, mechanical property, polypropylene

## Abstract

The effects of the ultra-high-molecular-weight (UHMW) component of polypropylene (PP) on its rheological properties, crystallization behavior, and solid-state mechanical properties were investigated using various measurement techniques. The terminal relaxation time—determined by measuring the linear viscoelasticity—was increased by adding the UHMW component. The increase in the melt elasticity produced by adding the UHMW component was observed by measuring the steady-state shear flow, although the shear viscosity was not greatly affected. Owing to the long characteristic time of the Rouse relaxation of the UHMW component, PP with the UHMW component formed highly oriented structures through a shear-induced crystallization process. The addition of the UHMW component enhanced the orientation and regularity of crystalline structure for extruded films. Therefore, the Young′s modulus, yield stress, and strength were higher in the PP film containing the UHMW component than in one without the UHMW component, irrespective of the direction of tensile deformation.

## 1. Introduction

Molecular weight and molecular weight distribution strongly affect various properties of polymeric materials [1] as well as branch structure [2,3] and the addition of nanofillers [4,5,6,7]. The physical and mechanical properties of polymeric materials such as viscosity, diffusion coefficient, drawability, and toughness are strongly influenced by the molecular weight and its distribution [8,9,10,11]. In particular, the processability and strength of a polymer are drastically improved by the addition of an ultra-high-molecular-weight (UHMW) component compared with samples with a monodispersed molecular weight distribution. Consequently, the influences of the UHMW component on the physical and mechanical properties of polymeric materials have been studied intensively [12,13,14,15,16].

The addition of UHMW chains significantly enhances strain hardening during the elongational flow process in various polymer melts [17,18,19,20]. The relaxation time of the Rouse mode (*τ*_R_), which corresponds to the characteristic time for chain contraction, strongly depends on the molecular weight described as *τ*_R_ ≅ *M*^2^. Therefore, the stretching of the UHMW chains cannot be relaxed during elongational flow. This results in significant strain hardening. Moreover, it is well known that UHMW chains form the “shish” of a shish–kebab structure. The shish–kebab structure is composed of extended-chain crystals (shish) and folded-chain crystals (kebab) grown from the shish. This shish-kebab structure often appears by flow-induced crystallization, because the stretching of the UHMW chains remains during the crystallization process [21,22]. The formation of the shish structure by UHMW chains has been observed by small-angle neutron scattering in blends of low-molecular-weight hydrogenated polyethylene and UHMW deuterated polyethylene [23,24]. Because the formation of the shish–kebab structure results in significant improvements in the modulus and strength [25,26], the addition of UHMW chains is one of the most effective methods of modifying the mechanical properties of semi-crystalline polymers. However, the effects of UHMW chains on the morphology, crystallization behavior, and mechanical properties of such polymers have been investigated separately. In particular, the effects of the addition of a UHMW component on the structure–mechanical properties relationship of the products obtained by a conventional processing operation such as film formation have not yet been elucidated.

In the present study, the effects of a UHMW component on the rheological properties, crystallization behavior, and mechanical properties of PP samples were systematically investigated. The Rouse time of UHMW chains is considerably longer than the characteristic time of the flow process, resulting in the enhancement of the shish formation and the promotion of a highly oriented structure. Included herein is a discussion of the effects of a UHMW component on the structure and mechanical properties of a solid-state PP film based on its rheological properties and crystallization behavior.

## 2. Experimental

### 2.1. Materials

A commercially available low-molecular-weight PP (LPP) having an unimodal molecular weight distribution (*M*_n_ = 0.43 × 10^5^, *M*_w_ = 3.1 × 10^5^, and *M*_z_ = 1.3 × 10^6^) with a melt flow rate (MFR) at 230 °C under 2.16 kgf of 7 g/10 min, and high-molecular-weight PP (HPP) having a bimodal molecular weight distribution (*M*_n_ = 0.33 × 10^5^, *M*_w_ = 7.2 × 10^5^, and *M*_z_ = 7.1 × 10^6^) with an MFR of 3 g/10 min, were acquired from Prime Polymer Co., Ltd. It should be noted that the melting temperatures of LPP and HPP were 161 and 163 °C, respectively, suggesting that the isotacticity of the PP samples were almost the same. LPP and HPP were blended with a weight fraction of HPP of 15 wt% using a 50 mm single-screw extruder at 220 °C. All samples included phosphate and hindered phenol compounds as antioxidants. The MFR of the blend was 6.3 g/10 min.

LPP and LPP/HPP films (approximately 25 μm thick) were prepared using a 75 mm single-screw extruder with a T-shaped die at 250 °C. The screw torques were 37% and 36% of the maximum for LPP and LPP/HPP, respectively. The die width was 600 mm, and the die gap was 0.8 mm. The extruded film was stretched in the air gap (10 mm) at 150 m min^−1^ by winding rolls. The chill rolls were maintained at 30 °C. The shear rate on the die wall ($\dot{\gamma_{\mathrm{wa}}}$), calculated using the following equation, was 590 s^−1^:

(1) $$\dot{\gamma_{\mathrm{wa}}}=\frac{6Q}{H^{2}W},$$

where *Q* is the volume flow rate, *H* is the die gap, and *W* is the width of the die.

The film density (*ρ*) values of LPP and LPP/HPP, which were measured using a density-gradient column at 23 °C, were 892 and 900 kg/m^3^, respectively.

Pellets of LPP and LPP/HPP were melt-pressed at 180 °C and 15 MPa for 5 min, then quenched at 25 °C to prepare sample sheets (approximately 0.8 mm thick) for melt-rheology measurements.

### 2.2. Measurements

The melt-state viscoelasticity values were measured using a stress-control rheometer (AR2000ex, TA Instruments, Inc., New Castle, DE, USA) equipped with a cone-and-plate system with a diameter of 25 mm and a cone angle of 4°. The frequency sweep was performed from 0.01 to 628.3 rad s^−1^ at 180, 200, and 230 °C under a nitrogen atmosphere. The storage and loss moduli (*G*′ and *G*″, respectively) measured at various temperatures were horizontally shifted to construct the master curves using time–temperature superposition. The reference temperature (*T*_r_) was set to 180 °C, which is above the melting temperature of PP samples. The shear stress and primary normal stress difference under a steady-state shear flow were also measured at different shear rates under the same conditions at 190 °C.

The transient elongational viscosity was measured using the stress-control rheometer with an extensional viscosity accessory (SER2-G, Xpansion Instruments, Tallmadge, OH, USA) under constant Hencky strain rates from 0.1 to 3.2 s^−1^ at 190 °C. Rectangular specimens (10 mm wide, 15 mm length, and 0.8 mm thick) prepared by compression molding were used for the measurements.

Capillary extrusion was performed using a capillary rheometer (140 SAS-2002, Yasuda Seiki Seisakusho, Ltd., Nishinomiya, Japan) at 190 °C to determine the steady-state shear viscosity. Circular-shaped dies with length (*L*)-to-diameter (*D*) ratios of 40/1 and 10/1 were used. The flow curve during the capillary extrusion was calculated using Bagley–Rabinowitsch corrections. The drawdown force defined as the force required to stretch a molten strand extrudate from a capillary rheometer [27] was evaluated using a tension detector (DT-413 G-04-3, Nidec-Shimpo, Kyoto, Japan) and a set of rotating rolls at 190 °C with draw ratios of 10 and 30.

**Figure 1:**
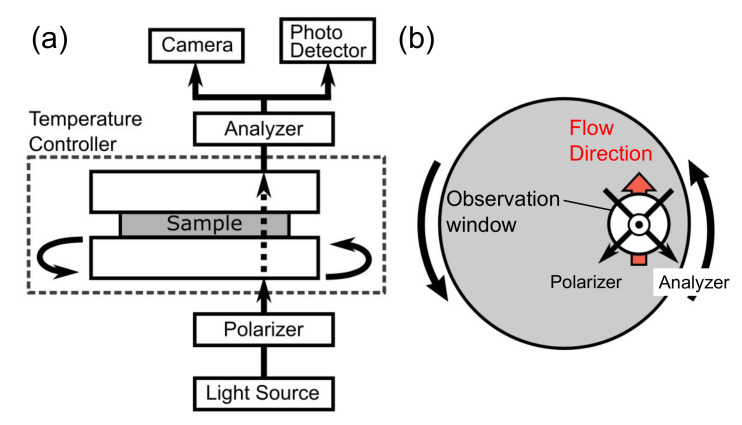
Schematics of the microscopic system combined with the shear stage viewed from (**a**) the side and (**b**) the top.

The crystallization behaviors of the samples with/without shear history were evaluated using a home-made machine comprising a polarized microscope (DM2700P, Leica Microsystems GmbH, Wetzlar, Germany), a shear stage with a temperature controller (CSS450, Linkam Scientific Instruments, Tadworth, Surrey, UK), and a photodetector (PM16-121, Thorlabs Inc., Newton, NJ, USA). A beam of light, which passed through the polarizer, the shear stage, and the analyzer, was directed into the photo detector, as shown in Figure 1. Parallel plates made of quartz were set in the stage, in which a circular window (2 mm in diameter) was provided to transmit the light. The center of this window was 10 mm from the center of the plate. A shear rate of 100 s^−1^, which was calculated from the rotation speed of the bottom plate and the gap between the plates, was applied. The angle between the flow direction in the plates and the transmission axis of the polarizer was π/4. After passing through the analyzer at the crossed polar configuration, the light intensity was measured by the photo detector mounted in one of the eyepieces of the polarized microscope. A camera was set at another eyepiece to observe the morphology of the samples. The light transmittance (*T*) was calculated as follows:

(2) $$T=\frac{I-I_{⊥}}{I_{\left|\right|}-I_{⊥}}$$

where *I*_⊥_ and *I*_||_ are the light intensities without a sample under crossed polars and parallel polars, and *I* is the light intensity passing through the sample under crossed polars.

The melting and crystallization temperatures were evaluated using a differential scanning calorimetry (DSC) system (DSC8500, PerkinElmer Co., Ltd., Waltham, MA, USA) under a nitrogen atmosphere. A 5 mg piece of each sample film was heated from 25 to 230 °C at a rate of 10 °C min^−1^ and was maintained at the isothermal temperature for 10 min. The sample was then cooled to 25 °C at a rate of 30 °C min^−1^. The crystallinity was calculated using the following equation:

(3) $$\chi_{\mathrm{DSC}}=\frac{\Delta H_{f}}{\Delta H_{f}^{0}},$$

where Δ*H*_f_ is the fusion enthalpy of the sample and Δ*H*_f_^0^ is the fusion enthalpy of a perfect sample of crystalline iPP (209 J g^−1^) [28]. It should be noted that the DSC curves were measured in the first heating and cooling processes to evaluate the influences of the morphology of film samples on the melting and crystallization behaviors. The rigid amorphous fraction (*χ*_RA_) was evaluated by temperature-modulated DSC measurements (step-scan mode) using the DSC8500 system. The step-scan procedure consisted of heating steps of 2 °C at 40 °C min^−1^ and isothermal steps of 0.4 min. The heat capacity during the heating process was obtained using step-scan software. The specific heat capacity increment at *T*_g_ (Δ*c*_p_) was estimated from the reversible specific heat capacity curve obtained from the step-scan measurement. *χ*_RA_ was calculated using the following equation [29,30]:

(4) $$\chi_{\mathrm{RA}}=1-\chi_{\mathrm{DSC}}-\frac{\Delta c_{p}}{\Delta c_{p,a}},$$

where Δ*c*_p,a_ is the specific heat capacity increment at *T*_g_ of the fully amorphous iPP. The value of Δ*c*_p,a_ was taken from the ATHAS database and from previous studies [31,32]. It should be noted that the value of *χ*_DSC_ obtained from the step-scan measurement was almost the same as that from the conventional measurement using Equation (3).

Two-dimensional wide-angle X-ray diffraction (WAXD) and small-angle X-ray scattering (SAXS) patterns of the sample films were measured using an XRD machine (SmartLab, Rigaku Corp., Akishima, Japan). A graphite-monochromatized Cu K*α* radiation beam (45 kV and 200 mA) was directed into the sample film, and the diffraction patterns were collected with a detector (HyPix-400, Rigaku, Corp., Akishima, Japan). The exposure time for both the WAXD and SAXS measurements was 15 min. The crystallinity of each film was calculated using the following equation:

(5) $$\chi_{\mathrm{WAXD}}=\frac{\sum_{i}I_{ci}}{\sum_{i}I_{ci}{+I}_{a}},$$

where *I*_c*i*_ and *I*_a_ are the integrated areas of the crystalline and amorphous peaks, respectively. The integrated area of each peak was obtained by fitting the sum of the Gaussian functions to the experimental integrated intensity profile. The Hermans’ orientation function (*f*_WAXD_) defined by the following equation was used to evaluate the orientation of the crystalline structure:

(6) $$f_{\mathrm{WAXD}}=\frac{{3\langle \cos}^{2}\varphi \rangle -1}{2},$$

where *φ* is the angle between the crystalline axis and the machine, i.e., the flow direction (MD). Based on the Wilchinsky method [33], $\langle \cos^{2}\varphi \rangle$ is described as:

(7) $$\langle \cos^{2}\varphi \rangle =1-1.090\langle \cos^{2}\phi_{110}\rangle -0.901\langle \cos^{2}\phi_{040}\rangle ,$$

where $\langle \cos^{2}\phi_{110}\rangle$ and $\langle \cos^{2}\phi_{040}\rangle$ are calculated using the following equation: [34,35]

(8) $$\langle \cos^{2}\phi_{hkl}\rangle =\frac{\int_{0}^{\pi }I\left(\phi_{hkl}\right)\cos^{2}\phi_{hkl}\sin\phi_{hkl}d\phi_{hkl}}{\int_{0}^{\pi }I\left(\phi_{hkl}\right)\sin\phi_{hkl}d\phi_{hkl}}.$$

Here, *I* (*ϕ_hkl_*) is the intensity distribution of the (*hkl*) plane at the azimuthal angle *ϕ*.

The polarized infrared (IR) spectrum of each film was measured using a Fourier-transform IR (FT-IR) spectrometer (Spectrum100, PerkinElmer Co., Ltd., Waltham, MA, USA) at 25 °C. The polarization direction of the incident laser was tuned to parallel and perpendicular to the MD using a polarizer. Each IR spectrum was accumulated 16 times with an exposure time of 2 s. The integrated intensity of each IR band was calculated by fitting the sum of the Voigt functions using Igor Pro software. The Hermans’ orientation functions of the crystalline and amorphous chains were calculated using the following equation [36,37]:

(9) $$f_{\mathrm{IR}}=\frac{2}{3\langle \cos^{2}\alpha \rangle -1}\frac{D-1}{D+2},$$

where *α* is the angle between the molecular chain axis and the transition moment of each molecular vibration, and *D* is the IR dichroic ratio defined as *D* = *A*_||_/*A*_⊥_, where *A*_||_ is the IR absorbance parallel to the stretching direction and *A*_⊥_ is the IR absorbance perpendicular to the stretching direction. In the present study, IR bands at 841 and 973 cm^−1^ assigned to the crystalline helical chains and the amorphous chains, respectively, were used to evaluate the orientation functions of the crystalline and amorphous chains (*f*_IR,c_ and *f*_IR,a_). It should be noted that the values of *α* of these IR bands are known to be 0° and 7.31° for 841 and 973 cm^−1^, respectively [37,38]. The crystallinity was calculated using the following equation: [39]

(10) $$\chi_{\mathrm{IR}}=0.614\frac{A_{998}}{A_{973}},$$

where *A*_998_ and *A*_973_ are the integrated intensities of the IR bands at 998 and 973 cm^−1^, respectively.

Dynamic mechanical analysis (DMA) was performed using a DMA machine (Rheogel-E4000, UBM Co., Ltd., Muko, Japan) to evaluate the temperature dependences of the loss and storage moduli (*E*′ and *E*″) in the tensile mode. The measurements were performed at 10 Hz in the temperature range from −80 to 160 °C at a constant heating rate of 2 °C min^−1^. Rectangular specimens (width 4 mm and length 10 mm), which were cut from the films, were used for the measurements. Oscillatory strain was applied parallel and perpendicular to the MD and the transverse direction (TD) of the films to evaluate mechanical anisotropy.

Tensile tests were performed using a tensile machine (EZ-LX HS, Shimadzu Corp., Kyoto, Japan) at a constant crosshead speed of 10 mm min^−1^ at 25 °C to evaluate the stress–strain behavior of the films. Dumbbell-shaped specimens (gauge length 10 mm, width 4 mm, and thickness approximately 25 μm) were cut from the films along the MD and TD and used for the measurements. Drawn specimens were prepared by the following procedure: each film specimen was drawn up to a strain of 1.5 and subjected to stress relaxation for 15 min; the drawn specimen was then removed from the tensile machine and kept for 1 day.

## 3. Results and Discussion

### 3.1. Rheological Properties

**Figure 2:**
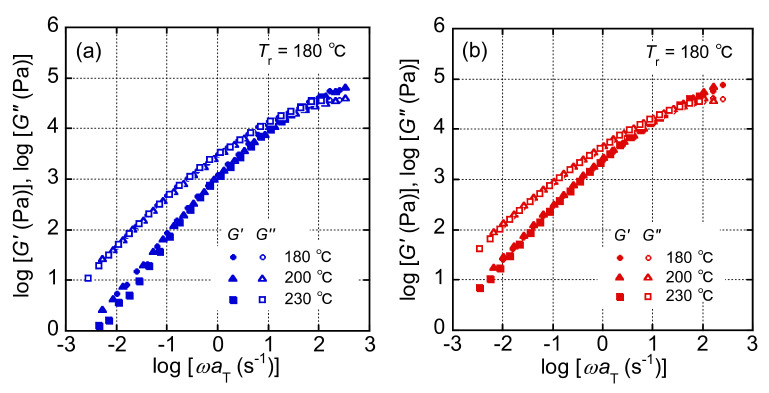
Angular−-frequency dependences of the storage and loss moduli (*G*′ and *G*″) of (**a**) LPP and (**b**) LPP/HPP (LPP is a unimodal PP and HPP is a bimodal PP). The reference temperature (*T*_r_) was 180 °C.

Figure 2 shows the angular frequency dependences of *G*′ and *G*″ of LPP and LPP/HPP. Both *G*′ and *G*″ decreased with decreasing the angular frequency and showed a cross point. The cross point for LPP/HPP was located at a lower frequency than that for LPP, suggesting that LPP/HPP has a longer relaxation time than LPP. Moreover, the modulus at the cross point for LPP/HPP was higher than for LPP. This is reasonable because LPP/HPP has a broad distribution of relaxation time. The slopes of *G*′ and *G*″ in the terminal relaxation region were almost 1 and 2, respectively. This is typical viscoelastic behavior of a simple polymer melt. However, the slopes of *G*′ and *G*″ in the low-frequency region of LPP/HPP were lower than those of LPP with an intense fashion of *G*′. This result was due to the long terminal relaxation time ascribed to the UHMW component of HPP. The broadening of the terminal relaxation region is a well-known effect of a UHMW component on rheological behavior [18]. Furthermore, the shift factors of both samples were almost the same, indicating both samples show a similar flow activation energy (≅45 kJ/mol).

**Figure 3:**
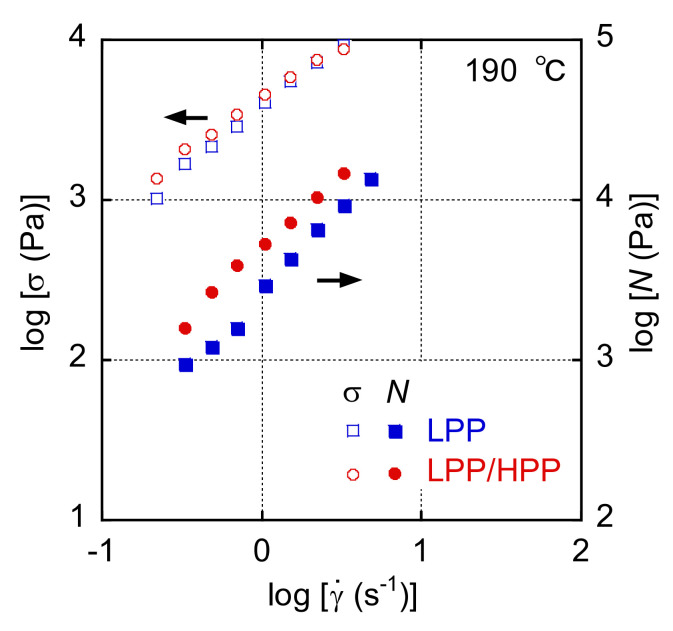
Shear-rate ($\dot{\gamma }$) dependences of the shear stress (*σ*) and the primary normal stress difference (*N*) of LPP and LPP/HPP at 190 °C (LPP is a unimodal PP and HPP is a bimodal PP).

The shear-rate ($\dot{\gamma }$) dependences of the shear stress (*σ*) and the primary normal stress difference (*N*) under steady-state shear flow are shown in Figure 3. Both *σ* and *N* increased with increasing $\dot{\gamma }$ for LPP and LPP/HPP. The values of *σ* were slightly higher for LPP/HPP than those for LPP in the low $\dot{\gamma }$ region, whereas the $\dot{\gamma }$ dependence of *σ* in the high $\dot{\gamma }$ region was almost the same for both PP samples. However, the values of *N* were higher for LPP/HPP than those for LPP over the whole $\dot{\gamma }$ region. This suggests that the increase in melt-state elasticity was caused by the addition of the UHMW component. These results corroborate those pertaining to linear viscoelasticity shown in Figure 2.

**Figure 4:**
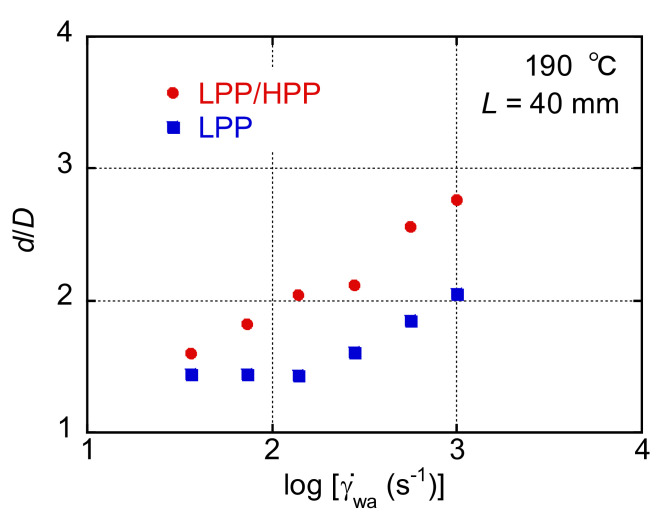
Ratio of the diameter of the extruded strand (*d*) and the die (*D*) plotted against the apparent wall shear rate (${\dot{\gamma }}_{\mathrm{wa}}$) during capillary extrusion.

Figure 4 shows the ratio of the diameter of the extruded strand (*d*) to the diameter of the die (*D*) plotted against the apparent shear rate on the wall (${\dot{\gamma }}_{\mathrm{wa}}$) when a die with a length of 40 mm was used. The *d*/*D* ratio increased monotonously as ${\dot{\gamma }}_{\mathrm{wa}}$ increased in both samples, and the *d*/*D* ratio of LPP/HPP was obviously higher than that of LPP. This is attributed to the enhanced primary normal stress difference with a prolonged relaxation time.

**Figure 5:**
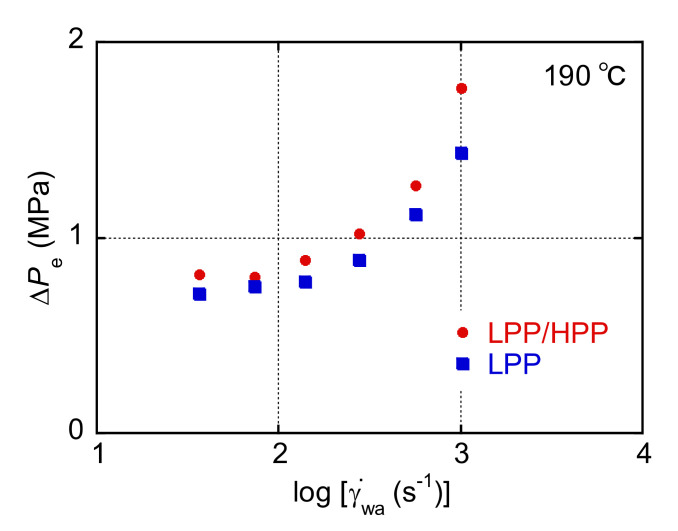
End-pressure drop (Δ*P*_e_) for LPP and LPP/HPP plotted against the apparent wall shear rate (${\dot{\gamma }}_{\mathrm{wa}}$) at 190 °C (LPP is a unimodal PP and HPP is a bimodal PP).

The end-pressure drop (Δ*P*_e_) at 190 °C evaluated using a Bagley plot is plotted against the apparent wall shear rate (${\dot{\gamma }}_{\mathrm{wa}}$) in Figure 5. Δ*P*_e_ increased with increasing ${\dot{\gamma }}_{\mathrm{wa}}$, which is typical capillary extrusion behavior. Moreover, the Δ*P*_e_ value of LPP/HPP was higher than that of LPP in the ${\dot{\gamma }}_{\mathrm{wa}}$ range, although *σ* was almost the same for both PPs, as shown in Figure 3. Considering that Δ*P*_e_ is determined by *σ* and *N* [40,41], this result is attributed to the enhancement of *N* by the addition of the UHMW component, as shown in Figure 3.

**Figure 6:**
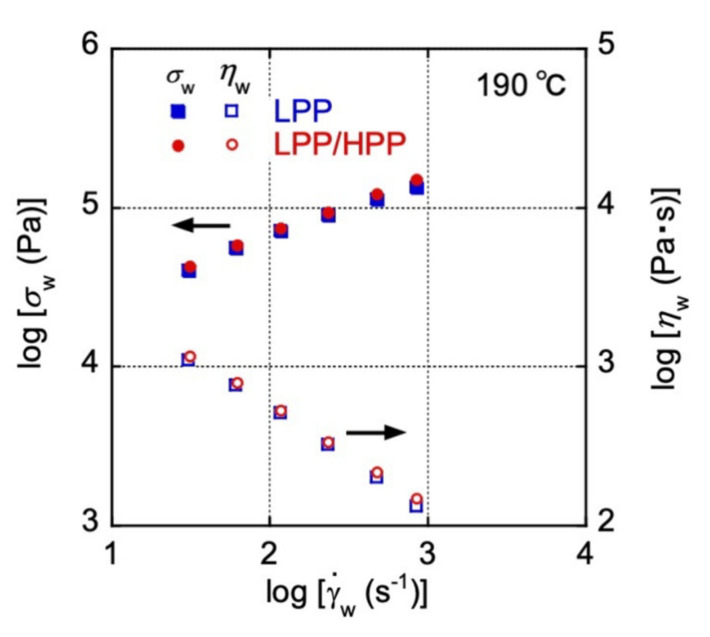
Steady-state shear stress (*σ*_w_) and viscosity (*η*_w_) plotted against the shear rate (${\dot{\gamma }}_{w}$) during capillary extrusion at 190 °C for LPP and LPP/HPP (LPP is a unimodal PP and HPP is a bimodal PP).

The ${\dot{\gamma }}_{w}$ dependences of *σ*_w_ and *η*_w_ after Bagley–Rabinowitsch corrections are shown in Figure 6. Both *σ*_w_ and *η*_w_ showed almost the same $\dot{\gamma }\cdot$ dependence for LPP and LPP/HPP, and the values of *σ*_w_ for LPP/HPP were very close to those for LPP, corroborating the results for *σ* in the high shear-rate region during steady-state shear flow shown in Figure 3. This result corresponds to the extrusion torque during T-die extrusion; both PP samples showed the same torque values.

**Figure 7:**
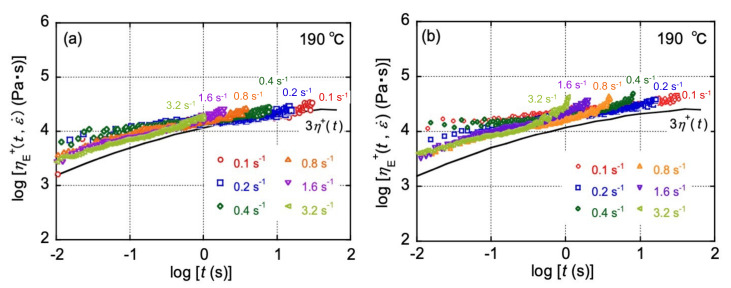
Transient uniaxial elongation viscosity (*η*_E_^+^(*t*, $\dot{\varepsilon }$)) at various Hencky strain rates ($\dot{\varepsilon }$) at 190 °C for (**a**) LPP and (**b**) LPP/HPP (LPP is a unimodal PP and HPP is a bimodal PP). The solid line represents the calculated viscosity growth curve.

The elongational viscosity growth curves at various strain rates under uniaxial elongational flow for both PP samples are shown in Figure 7. The solid line in Figure 7 represents 3*η*^+^(*t*) calculated from *G*′ and *G*″ shown in Figure 2 using the following equation [42,43]:

(11) $$\eta^{+}\left(t\right)=t{\left.\left[G^{\prime \prime }\left(\omega \right)+1.12G^{\prime }\left(\frac{\omega }{2}\right)-0.200G^{\prime }\left(\omega \right)\right]\right|}_{\omega \to \left(1/t\right)}.$$

The upward deviation of *η*_E_^+^(*t*, $\dot{\varepsilon }$) from 3*η*^+^(*t*) suggests the strain hardening. LPP showed almost no strain hardening during elongational flow because the flow curves were close to 3*η*^+^(*t*) at all strain rates. On the other hand, LPP/HPP showed weak strain hardening at the strain rates from 0.4 to 3.2 s^−1^, indicating that strain hardening was promoted by the stretching of the UHMW chains. The promotion of strain hardening by the addition of UHMW chains has been reported for various polymeric materials [17,18,19,20,44]. It has been well known that the strain hardening is responsible for good processability at various processing operations. For example, the stability of tubular blown film is improved greatly, which makes it possible to produce a thin film [45,46].

The values of the drawdown force during the capillary extrusion of both PP samples at draw ratios (DRs) of 10 and 30 are listed in Table 1. At both draw ratios, the values of the drawdown force were considerably higher for LPP/HPP than those for LPP. The markedly high drawdown force for LPP/HPP should be attributed to strain hardening during uniaxial elongation at the high strain rate, as shown in Figure 7, and/or the strain-induced crystallization of the UHMW chains [7,27].

### 3.2. Crystallization Behavior

**Figure 8:**
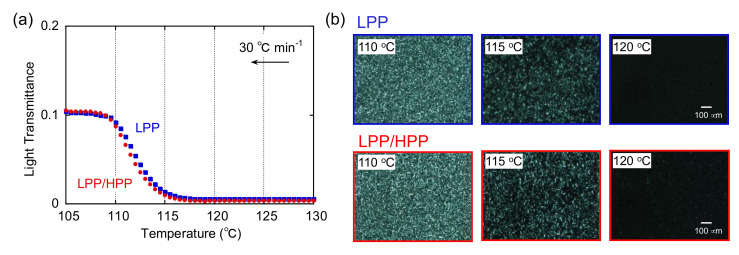
(**a**) Temperature dependence of light transmittance and (**b**) polarized optical microscopy (POM) images at 110, 115, and 120 °C during non-isothermal crystallization at 30 °C min^−1^ in the absence of shear flow for both polypropylene (PP) samples.

The temperature dependence of light transmittance during non-isothermal crystallization without shear flow is shown in Figure 8. Each sample was initially heated to 230 °C for 10 min to erase its thermal history. The samples were then cooled at 30 °C min^−1^, i.e., the same rate used for the DSC measurements. In each sample, the light intensity increased drastically at approximately 117 °C and reached an asymptotic value at below 107 °C, in good agreement with the DSC results discussed later, i.e., both PP samples had almost the same crystallization temperature at approximately 117 °C. Moreover, spherulites were observed in the polarized optical microscopy (POM) images of both PP samples at below 117 °C. These results are typical crystallization behavior for a semi-crystalline polymer in the absence of shear flow [47,48].

**Figure 9:**
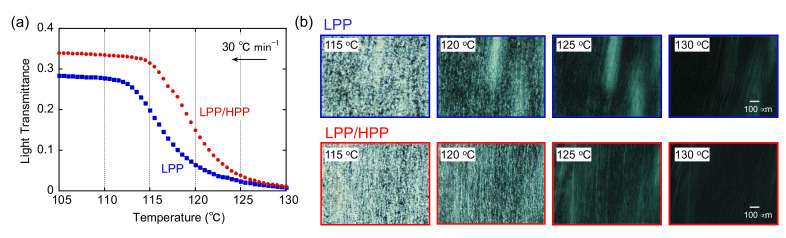
(**a**) Temperature dependence of light transmittance and (**b**) polarized optical microscopy (POM) images at 115, 120, 125, and 130 °C during non-isothermal crystallization at 30 °C min^−1^ after the cessation of shear flow (100 s^−1^) at 160 °C.

Figure 9 shows the temperature dependence of light transmittance after exposure to shear flow during cooling from 230 to 160 °C. The shear rate was 100 s^−1^. After the cessation of shear, the samples were cooled at the same rate. Therefore, the applied thermal history was the same as in Figure 8. Although it is not clear from the figure, light transmittance was unchanged at 160 °C, suggesting that flow-induced birefringence in the molten state was negligible. The onset crystallization temperature after the applied shear flow was obviously higher than that in the absence of shear for both PP samples, which is consistent with previous results of the flow-induced crystallization of PP [47]. Moreover, streak-like patterns caused by molecular orientation in the flow direction were observed in the POM images at 125 °C for both samples. These results indicate that the oriented molecular chains acted as nuclei for the formation of the shish-like structure, resulting in enhanced crystallization compared with crystallization in the absence of shear flow. When crystallization was almost completed (<115 °C), both highly oriented crystals and spherulites were observed in the POM image of LPP. However, highly oriented structure such as the shish–kebab structure was clearly observed in the LPP/HPP sample, owing to the strong orientation of the UHMW chains. The strong orientation of the molecular chains of LPP/HPP compared with those of LPP is consistent with the marked light transmittance below 115 °C for LPP/HPP. Reduced light scattering originating from the spherulite texture as well as a long correlation length resulted in high light transmittance.

The Weissenberg number associated with the Rouse mode (Wi_R_), defined as ${\mathrm{Wi}}_{R}={\dot{\gamma }\tau }_{R}$, is used as a critical factor for forming flow-induced crystalline structure [22]. It is known that the flow-induced crystalline structure is formed at which Wi_R_ is larger than unity. The Wi_R_ values of both PP samples for the flow-induced crystallization process were calculated using the following equation [49]:

(12) $${\mathrm{Wi}}_{R}={\dot{\gamma }\tau }_{e}{\left(\frac{M}{M_{e}}\right)}^{2},$$

where *τ*_e_ is the Rouse time of an entanglement strand and *M*_e_ is the entanglement molecular weight. For iPP, Wi_R_ was calculated using *τ*_e_ = 1.5 × 10^−7^ s and *M*_e_ = 5.25 kg mol^−1^ at 170 °C [50]. In the case of the present crystallization process, the critical molecular weight for flow-induced crystallization was approximately 1.3 × 10^6^, calculated using Equation (12). Considering that both PP samples included a small number of UHMW chains, the highly oriented crystalline structure observed in the POM image shown in Figure 9 was caused by stretching of the UHMW component. In particular, the LPP/HPP sample included a larger amount of the UHMW component than the LPP sample, which resulted in a significant degree of orientation in the flow direction and numerous highly oriented crystals in LPP/HPP compared with LPP.

### 3.3. Morphology and Mechanical Properties of Films

**Figure 10:**
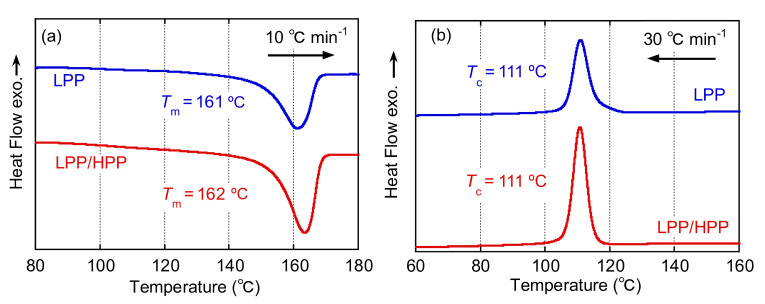
Differential scanning calorimetry (DSC) curves during (**a**) heating at 10 °C min^−1^ and (**b**) cooling at 30 °C min^−1^ for LPP and LPP/HPP (LPP is a unimodal PP and HPP is a bimodal PP).

The DSC curves obtained during the heating and cooling processes of LPP and LPP/HPP films at heating and cooling rates of 10 and 30 °C min^−1^, respectively, are shown in Figure 10. The melting and crystallization temperatures (*T*_m_ and *T*_c_) were almost the same for both PP films, as denoted in Figure 10, which is in good agreement with the crystallization behavior demonstrated by the light transmittance shown in Figure 8. Moreover, as shown in Table 2, both PP samples had almost the same crystallinity, whereas the amount of rigid amorphous structure in LPP was higher than in LPP/HPP, suggesting that the LPP film included a large amount of imperfect crystalline structure compared with LPP/HPP.

**Figure 11:**
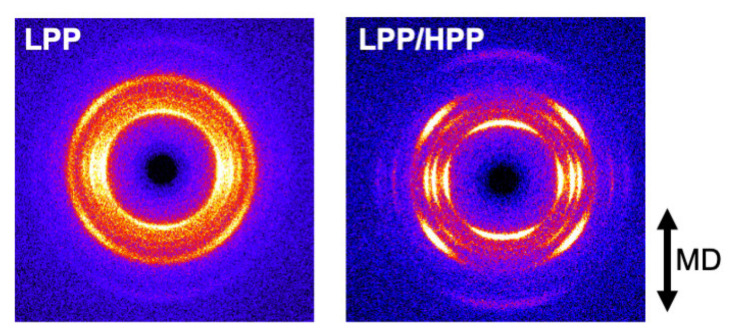
Two-dimensional wide-angle X-ray diffraction (WAXD) patterns of LPP (**left**) and LPP/HPP (**right**) (LPP is a unimodal PP and HPP is a bimodal PP).

**Figure 12:**
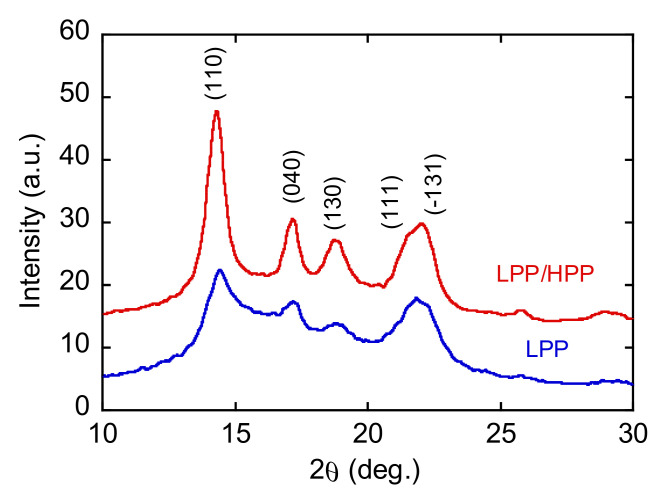
Integrated intensity profiles plotted against 2*θ* for LPP and LPP/HPP (LPP is a unimodal PP and HPP is a bimodal PP).

The two-dimensional WAXD patterns of the LPP and LPP/HPP films are shown in Figure 11. Each PP film showed five diffraction peaks assigned to (110), (040), (130), (111), and (−131) of the PP *α*-crystals [51]. The integrated intensity profiles are plotted against 2*θ* in Figure 12. The figures in parentheses are the Miller indices. The crystalline diffraction peaks of LPP/HPP were sharper and stronger than those of LPP, although the crystallinity values calculated using Equation (3) were similar for each sample, as shown in Table 2. These results indicate that the crystalline regularity of LPP was imperfect compared with that of LPP/HPP. This corroborated the DSC results, which demonstrated that LPP included more rigid amorphous structure than HPP/LPP. It should be noted that the crystallinity determined by IR spectroscopy listed in Table 2 was similar for both PP samples, as corroborated by the DSC and WAXD results.

**Figure 13:**
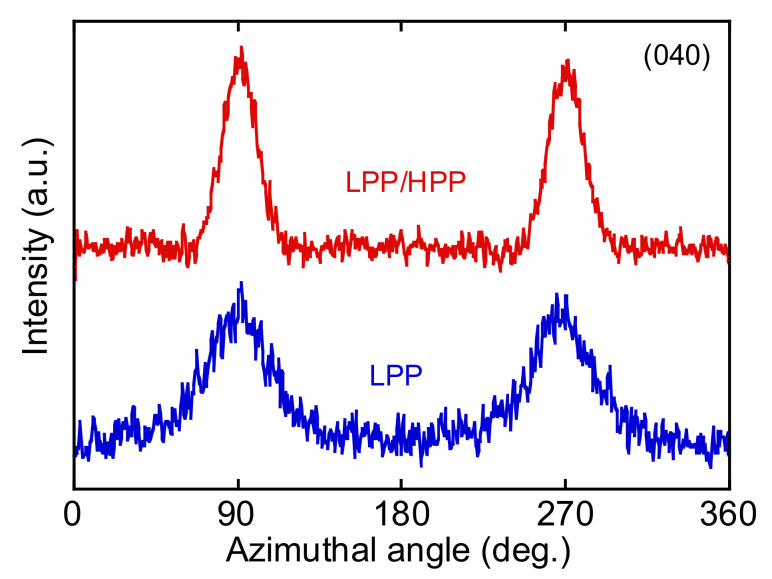
Azimuthal-angle distribution of the (040) diffraction plane of LPP and LPP/HPP *α* crystals (LPP is a unimodal PP and HPP is a bimodal PP).

The azimuthal-angle dependence of the integrated intensity of the (040) diffraction peak is shown in Figure 13. There were sharp peaks in the intensity distribution at 90° and 270°, corresponding to the equator axis in Figure 11, for both PPs, suggesting that the c axis of the crystalline structure was oriented to the MD. The orientation of the crystalline structure was enhanced by the addition of the UHMW component because the peak width of the intensity profile of LPP/HPP was sharper than that of LPP. The orientation function values evaluated by WAXD and IR spectroscopy shown in Table 2 also suggest the enhancement of the crystalline structure orientation due to the addition of the UHMW component.

**Figure 14:**
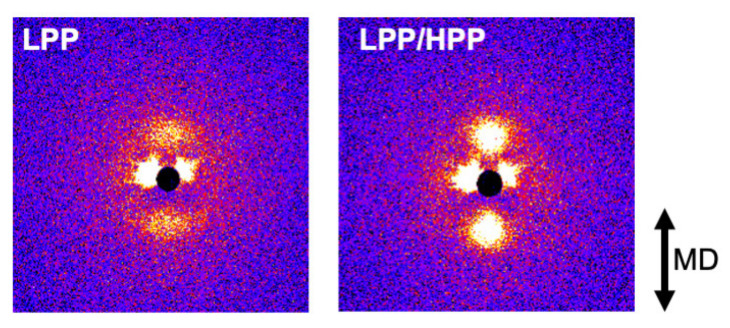
Two-dimensional small-angle X-ray scattering (SAXS) patterns of sample films of LPP (**left**) and LPP/HPP (**right**) (LPP is a unimodal PP and HPP is a bimodal PP).

The two-dimensional SAXS patterns of the oriented films are shown in Figure 14. LPP/HPP exhibited a strong two-peak pattern in the MD compared with LPP, indicating that the addition of the UHMW component enhanced the lamellar orientation to the perpendicular direction of the MD. Such lamellar orientation behavior is corroborated by the WAXD results.

According to the evaluation of the rheological and crystallization behaviors of LPP and LPP/HPP, the strong orientation of the UHMW chains to the MD should remain during film processing, which results in the improvement of the orientation of the lamellar crystalline structure by the addition of the UHMW component.

**Figure 15:**
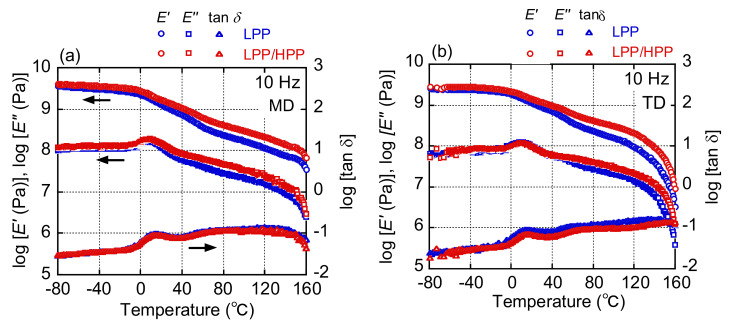
Temperature dependence of the storage modulus (*E*′), loss modulus (*E*″), and loss tangent (tan*δ*) at 10 Hz for LPP and LPP/HPP at the applied strain along (**a**) the machine direction (MD) and (**b**) the transverse direction (TD) (LPP is a unimodal PP and HPP is a bimodal PP).

The dynamic mechanical spectra of LPP and LPP/HPP films measured in the MD and TD are shown in Figure 15. Strain was applied along the MD and TD to investigate the effects of molecular orientation on solid-state viscoelasticity. For both PP samples, the *E*′ and *E*″ values for MD stretching were higher than those for TD stretching owing to the orientation to the MD. Moreover, for both MD and TD stretching, the *E*′ and *E*″ values for LPP/HPP were higher than those for LPP above the glass transition temperature at approximately 20 °C. These results were caused by the imperfect crystalline structure of LPP compared with that of LPP/HPP; disorder in the crystalline structure leads to low thermal stability.

**Figure 16:**
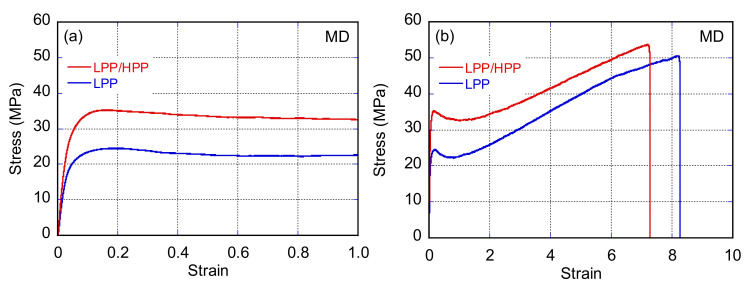
Stress–strain curves (**a**) in the yielding region and (**b**) up to the strain at break of LPP and LPP/HPP films stretched along the flow direction (MD) (LPP is a unimodal PP and HPP is a bimodal PP).

**Figure 17:**
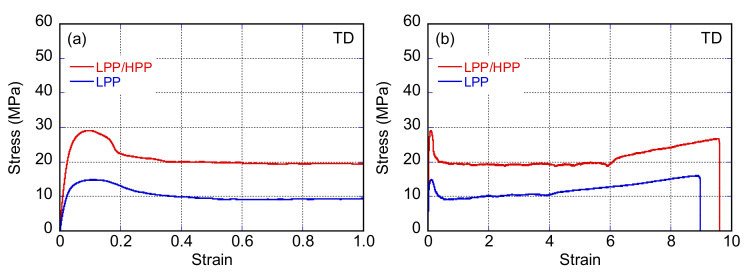
Stress–strain curves (**a**) in the yielding region and (**b**) up to the strain at break of LPP and LPP/HPP films stretched along the transverse direction (TD) (LPP is a unimodal PP and HPP is a bimodal PP).

**Figure 18:**
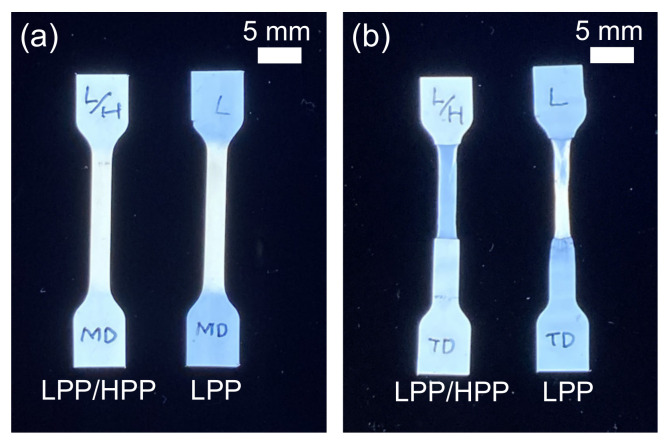
Polarized optical microscopy (POM) images of drawn specimens of LPP and LPP/HPP elongated up to a strain of 1.5 along (**a**) the flow direction (MD) and (**b**) the transverse direction (TD) (LPP is a unimodal PP and HPP is a bimodal PP).

Figure 16 and Figure 17 show the stress–strain curves of LPP and LPP/HPP films stretched along the MD and TD, respectively. The Young’s modulus, yield stress, and strength of LPP/HPP were higher than those of LPP with regard to MD stretching. As shown in Figure 18, the drawn specimens elongated up to *ε* = 1.5 along the MD exhibited homogeneous birefringence color without necking formation. Moreover, the two-dimensional WAXD patterns of the LPP and LPP/HPP films were almost the same as those of the undrawn films shown in Figure 11, suggesting that the initial crystalline structure of the undrawn films remained during the stretching process. These results indicate that the yielding deformation associated with MD stretching was caused by the pull-out of the molecular chains from the crystalline structure [52,53], because the pull-out deformation is homogeneously caused in all lamellar crystals. This is consistent with the homogeneous deformation without necking formation as observed in Figure 18a. Consequently, the improvements in the Young’s modulus, yield stress, and strength following the addition of the UHMW component resulted from the higher molecular orientation and high regularity of the crystalline structure.

**Figure 19:**
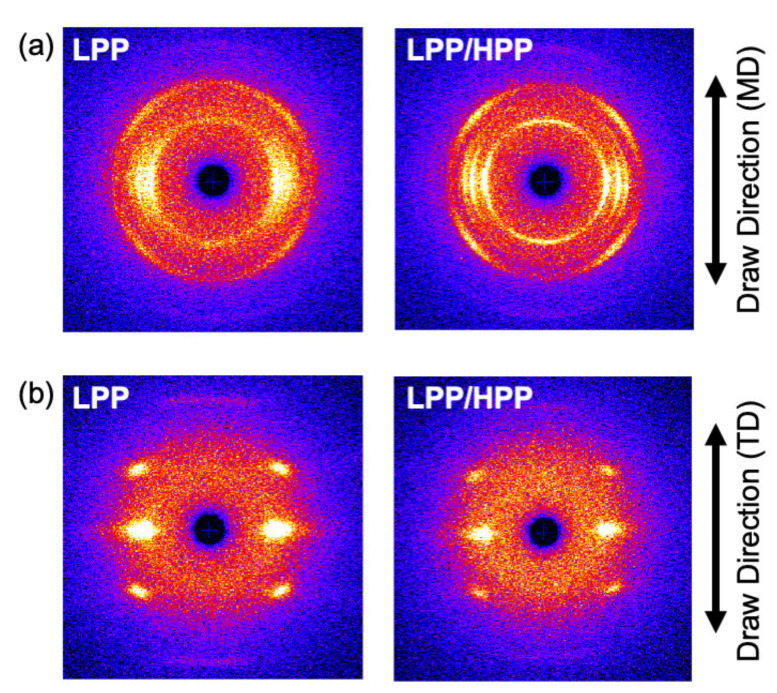
Two-dimensional wide-angle X-ray diffraction (WAXD) patterns of drawn LPP (left) and LPP/HPP (right) elongated along (**a**) the flow direction (MD) and (**b**) the transverse direction (TD) (LPP is a unimodal PP and HPP is a bimodal PP).

With regard to TD stretching, the Young’s modulus, yield stress, and strength of LPP/HPP were increased by the addition of the UHMW component, as with MD stretching. However, in contrast to MD stretching, the stress–strain curve associated with TD stretching for each PP sample featured a neck-propagation region, and clear necking occurred in the drawn specimens of both samples elongated in the TD, as shown in Figure 18b. The two-dimensional WAXD patterns of the drawn specimens shown in Figure 19b suggest that the crystalline structure was highly oriented in the TD direction, and the orientation degree of LPP/HPP was higher than that of LPP. Moreover, the crystalline structure associated with TD stretching was significantly disordered in both samples because the diffraction peaks were obviously broadened. These results suggest that the initial crystalline structure that was highly oriented along the MD fragmented into smaller crystallites and reoriented in the TD direction, leading to the formation of necking [54,55,56]. The crystalline structure of the undrawn LPP film was disordered compared with that of the undrawn LPP/HPP, as shown in Figure 11. Therefore, the Young’s modulus, yield stress, and strength of LPP/HPP were higher than those of LPP in the TD stretching. It should be noted that the orientation behavior of the drawn specimens of LPP and LPP/HPP evaluated by WAXD measurements were identical to those evaluated by birefringence measurements.

## 4. Conclusions

The present study comprised an investigation of the effects of a UHMW component on the rheological properties, crystallization behavior, and mechanical properties of PP. The elasticity of the melt state was improved by adding the UHMW component, as demonstrated by the higher values of the primary normal stress difference, the extrudate swell ratio, and the drop in end pressure of LPP/HPP than of LPP. It should be noted that the enhancements in rheological properties resulting from the addition of the UHMW component were achieved without an increase in the shear viscosity. Moreover, LPP/HPP exhibited strain hardening during uniaxial elongational flow in the high strain rate region. The strain hardening was due to that the orientation of the UHMW chains cannot be relaxed during uniaxial elongation owing to the characteristic long duration of the Rouse mode. With regard to the crystallization behavior, the addition of the UHMW component had no influence on the crystallization temperature, and spherulites were formed in both PP samples. However, the formation of a highly oriented structure was enhanced by the strong orientation of the UHMW chains during crystallization with shear flow. The enhancement of the molecular orientation by the addition of the UHMW component was also observed in the T-die films. Moreover, the numbers of crystalline defects and rigid amorphous structures decreased following the addition of the UHMW component, resulting in the strong orientation of crystalline structure in LPP/HPP compared with in LPP. Consequently, the Young’s modulus and strength of the LPP/HPP films were higher than those of the LPP films for both MD and TD stretching. These results provide useful information for the improvement of the rheological and mechanical properties of semi-crystalline polymeric materials.

## Figures and Tables

**Table 1:** Drawdown force during the capillary extrusion of polypropylene (PP) samples at draw ratios (DRs) of 10 and 30.

Sample Code	Drawdown Force (mN)	
	DR = 10	DR = 30
LPP	56	70
LPP/HPP	86	96

**Table 2:** Characteristics of the LPP and LPP/HPP films (LPP is a unimodal PP and HPP is a bimodal PP).

Sample Code	*χ*_DSC_ (wt%)	*χ*_RA_ (wt%)	*χ*_WAXD_ (wt%)	*χ*_IR_ (wt%)	*f*_WAXD_	*f*_IR,c_	*f*_IR,a_
LPP	34.3	59.5	49.3	51.8	0.12	0.22	0.15
LPP/HPP	34.5	54.1	52.1	53.6	0.18	0.28	0.16

## Data Availability

Not applicable.
